# Comparing efficiency of literature-based estimation of general biomass using Length-Weight Relationships (LWRs) between freshwater and seawater fishes

**DOI:** 10.1371/journal.pone.0321571

**Published:** 2025-06-03

**Authors:** Jaehoon Yeom, Sang Don Kim

**Affiliations:** 1 School of Environment and Energy Engineering, Gwangju Institute of Science and Technology, Gwangju, Republic of Korea; The Islamia University of Bahawalpur Pakistan, PAKISTAN

## Abstract

Fish biomass estimation is crucial for understanding aquatic ecosystem dynamics and managing fisheries resources effectively. This study evaluated the applicability of estimating fish biomass in Korean aquatic ecosystems using literature-derived General Total Length (GTL) and length–weight relationships (LWRs). We compared the estimated biomasses with measured field biomasses from literatures for freshwater and seawater fish species. Data on individual biomass, habitat, and length-weight coefficients were collected from the literature and databases for 245 fish species. Biomass was estimated using the GTL and representative LWR coefficients and then they were compared to the measured field biomasses from literature. The results showed that this estimation method was applicable only to freshwater species (R² =  0.7133), whereas the estimates for seawater species showed a poor correlation (negative R² values). The removal of outliers (Q >  6) improved the estimation accuracy for the freshwater species. This study demonstrates that literature-based biomass estimation using GTL and LWRs is appropriate for freshwater fish in Korea, but not for seawater species. These findings contribute to the generation of fundamental biomass data for ecosystem modeling and highlight the need for habitat-specific approaches for biomass estimation. Since habitat specific biomass data is deficient, future research should explore biomass estimation through species extrapolation to address data gaps in aquatic ecological studies.

## Introduction

Recently, concerns have arisen regarding the aquatic ecology of freshwater and saltwater ecosystems, with respect to global biodiversity. Aquatic ecosystems, including freshwater and saltwater, act as habitats for approximately 20% of all species on Earth [1–3]. Therefore, aquatic environments have long been the primary focus of ecological studies. Consequently, research on aquatic ecosystems has been continuously conducted over extended periods across various regions, primarily focusing on ecosystem monitoring, community structure analyses, life cycle studies, and ecosystem changes [4,5]. In addition, progress in ecological modeling has encouraged scientists to analyze ecological phenomena using mathematical and simulation-based systems [6–8].

Recent advancements in machine learning techniques have led scientists to explore the application of machine learning in ecological modeling [9]. Despite advancements in methodologies, limited accessibility to essential information and parameters for ecological research has resulted in data gaps in several regions. This hinders the development of ecological models based on big data and their applications in real-world environments [10–13]. Supplementation of insufficient ecological datasets and reliable processing of existing databases is crucial for advancing long-term aquatic ecosystem research. Therefore, to promote the development of long-term studies on aquatic ecosystems, it is essential to address the gaps in ecological data while ensuring the accurate and trustworthy handling of existing datasets [14].

Biomass is a fundamental parameter used in various ecological modeling studies. Therefore, accurate information of the biomass of organisms living within ecosystems is essential in many areas of ecological research [15–17]. However, the complex structure of aquatic ecosystems makes it difficult to estimate the biomass of the organisms living within different habitats. The biomass of organisms in an ecosystem is typically estimated by either inferring the biomass from compiled environmental data or converting easily measurable parameters into biomass [15,18,19]. The biomass of aquatic organisms in an aquatic ecosystem is typically estimated through a regression analysis between body length and weight, known as the length–weight relationships (LWRs) [15,20–22]. LWRs can be widely applied to aquatic invertebrates and fish, and estimations using this regression method are known to be highly accurate [15,21–24]. Therefore, when the direct measurement of mass is not possible, using LWRs to approximate the biomass of a species is a suitable approach. Therefore, estimating the weight of species that are difficult to measure directly through the proper application of length-weight relationships is a crucial methodology in ecosystem research. However, LWRs are not universally applicable to all species, and LWR coefficients can vary depending on the age, sex, maturity, food availability, parasitism and condition factor, leading to different estimates for the same species [20,25–29]. This is attributed to nutritional changes in fish caused by seasonal variations and different life stages [27,30]. In general, these differences can be evaluated using condition factors [20,31].

The studied area, Korea is surrounded by the sea on three sides and has four major rivers running through the country. As a result, both freshwater and saltwater regions coexist in Korea, with estuarine areas forming downstream of freshwater systems. Owing to these geographical characteristics, many fish species in Korean aquatic ecosystems inhabit both freshwater and seawater, alternating between the two environments. In addition, some species live exclusively in freshwater or seawater [32,33]. Fish species in Korea are distributed across various habitats, including freshwater, seawater, estuaries, and mudflats, and each species occupies a distinct environment. The habitats of these species have been identified in various preliminary aquatic ecology studies conducted in Korea. Therefore, when studying Korean aquatic ecosystems including LWRs research, it is crucial to account for these environmental factors, because Korea hosts a wide range of coexisting aquatic environments [34,35].

Although there is a lack of comprehensive information regarding the coefficients of length-weight relationships (LWRs) of fish species living in Korea, LWRs of freshwater and saltwater fish species in the Korean aquatic environment have been analyzed in several studies [36,37]. However, comparative studies of LWR between fish species known to inhabit freshwater and saltwater have not been conducted much. In addition, Korean ecologists and fishery scientists have collected quantified samples of numerous species, documented the lengths of fish species in web databases, and compiled field guides since the 1980s. Therefore, the generalized body length of fish species can be assessed using these literature sources [38,39].

This study focused on developing a methodology to estimate the generalized biomass of individual fish using length-weight relationships for fish species inhabiting Korea, thereby contributing to fields such as aquatic ecological modelling.

## Method

### Collecting monitored individual biomass of fish species in aquatic ecosystem in Korea including freshwater and seawater

**Fig 1:**
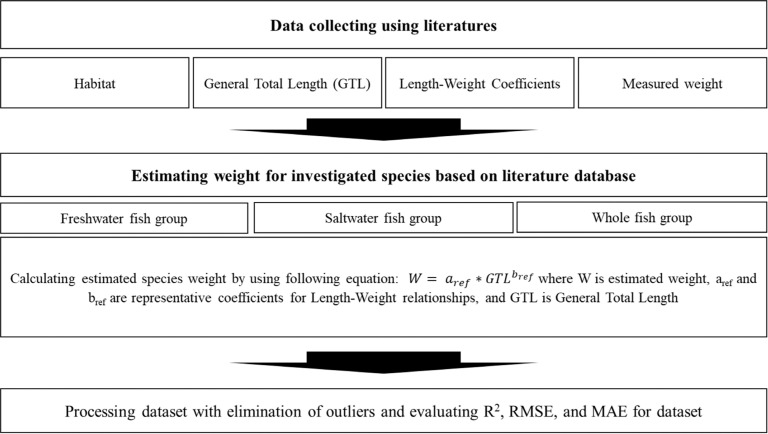
Scheme of methodology for whole study process.

Recorded individual biomass data for each species living in Korean aquatic ecosystems were collected from the literature on Korean aquatic ecosystems, including research articles, theses, and domestic conference papers (Fig 1). All datasets from the literature were included without any limitations on the investigation period, site, or species. However, monitoring data from the sea far from the mainland is excluded.

Biomass data from the collected literature are typically reported as the number density and mass density. To calculate the individual biomass of each species, biomass density was divided by number density using the following formula:


$$\text{Individual biomass }\left(\text{g}\right)=\frac{Biomassdensity\left(g/m^{2}\right)}{numberdensity\left(/m^{2}\right)}$$ (1)

The individual biomass for each species was calculated separately for each dataset organized by month. Individual biomass values calculated for each species were recorded as separate data points.

### Evaluating general total body length (GTL) and habitat of each species by using literature studies

General Total Body Length (GTL) was used as the representative body length for each species and was calculated based on averages determined by experts, field guides, or reliable databases. This study assumed that GTL represents the average length of all individuals of a species inhabiting Korean freshwater ecosystems.

The GTL for each species was derived from literature sources, including web databases, such as FishBase, online government databases, and field guides [40]. The GTL was calculated through a series of steps.

First, meta-web databases such as FishBase were used to estimate the GTL for the target species [40]. For species found in Korea, GTL was calculated by averaging the minimum and maximum lengths recorded in the datasets specifically related to the Korean region. Datasets with small sample sizes (n <  20) were excluded from analysis.

Second, for species lacking data in the meta-databases, GTL was determined using online and offline field guides that provided general species information.

The habitats of the target species were assessed by reviewing the literature, including government publications, domestic field guides, and online databases. The habitats of all the fish species mentioned in the studies were categorized into four groups: freshwater, seawater, mudflats, and estuaries. Species occupying multiple habitats were also recorded. This study aimed to identify species restricted to freshwater, species restricted to seawater, and species that inhabited mixed habitats.

### Selecting freshwater and seawater fish species and collecting length-weight relationship coefficients

Species that inhabit freshwater and seawater were selected from the database. The species were classified into three groups: ‘Only Freshwater,’ ‘Only Seawater,’ and a combined group labeled ‘Whole Species,’ which included both freshwater and oceanic species. The length-weight coefficients were collected for each group using a web database.

Because the target species were based on biomass studies conducted in Korea, we utilized length-weight coefficients from studies that focused on species inhabiting Korean waters. These constants were primarily gathered for data from Korean studies that were used in the FishBase web database. When multiple constants were available for a species from the studies conducted in Korea, a representative constant was calculated for each species. The representative coefficients a and b for the LWRs were calculated using the following formulae:


$$representativea=\text{Geometric mean of a}=\sqrt[{n}]{\prod_{k=1}^{n}a_{k}}$$ (2)

$$representativeb=\text{Arithmetic mean of b}=\frac{\sum_{k=1}^{n}b_{k}}{n}$$ (3)

### Biomass estimation and accuracy assessment

The representative weight of each species was calculated using the Length-Weight Relationship (LWR) coefficients and the General Total Length (GTL) recorded for each fish species. The estimated weight of each species was derived using the corresponding length-weight equations.

The concordance of the estimated general biomass of each species was evaluated using the coefficient of determination (R²) for the y =  x regression, in which the field biomass measured dataset (x) from literatures (x) was compared with the estimated dataset (y). The equation for the coefficient of determination is defined as $R^{2}=1-\frac{\sum_{i=1}^{n}{\left(y-\hat{y}\right)}^{2}}{\sum_{i=1}^{n}{\left(y-\bar{y}\right)}^{2}}$, where y represents the recorded values of individual biomasses from literatures for each species, $\hat{y}$ is the estimated value, and $\bar{y}$ is mean of the field measured biomasses from literatures. The coefficient of determination (R2) quantifies the proportion of variance in the estimated dataset that matches the field biomass dataset (x) from literature for an individual biomass.

In the regression analysis, the outliers were excluded using the Q value, which was calculated based on the residuals between the recorded data from field and estimated data. The Q value is defined as $Q_{i}=\frac{\left|y_{i}deviation\right|}{MedAD}$ where Q_i_ is the score used to identify outliers, y_i_ is the monitored biomass for each species, and MedAD is the median absolute difference between the estimated and monitored weights [41]. In this study, data points with Q >  6 were excluded to mitigate the influence of outliers.

Cook’s distance was used to identify and eliminate influential data points in the regression analysis. Cook’s distance is a measure used in regression analysis to identify influential data points. The formula for Cook’s distance D_i_ for the i-th observation is given by $D_{i}=\frac{\sum_{j=1}^{n}{\left({\hat{y}}_{j}-{\hat{y}}_{j\left(i\right)}\right)}^{2}}{p\cdot MSE}$. where ${\hat{y}}_{j}$ is the predicted value for the jth observation with all the data points included, ${\hat{y}}_{j}$ is the predicted value for the jth observation with the ith data point excluded, p is the number of predictors (including the intercept), and MSE is the mean-squared error of the regression model [42]. Ordinary least squares regression was applied to the dataset after the removal of these outlier points to ensure the accuracy of the analysis.

The calculated representative weight for each species was compared with the actual recorded weight from the field data to determine the method that produced the most accurate estimates. The accuracy of the estimated values was quantitatively compared across different groups using R² values.

## Results

### Results of literature-based monitoring data, habitat classification, and collection of length-weight coefficients

The compiled dataset for the measured field biomasses from literatures of fish species in Korea was obtained from various sources [43–49]. The detailed habitat composition of the compiled dataset is presented in Table 1. Species in the dataset were classified into four categories: species inhabiting only seawater and mudflats; species inhabiting only freshwater; species inhabiting both freshwater and seawater or estuaries; and species with no recorded habitats (Table 1). In this study, species groups living exclusively in seawater and mudflats, species living only in freshwater, and a combined group of all species from both categories were used to evaluate the accuracy of the proposed methodology.

**Table 1:** Number of Fish Species in Korea by Habitat Based on Literature Review.

Habitat name	Only seawater fish and mud	Only freshwater fish	Mixed area	No record	Total
Number of species	51	55	48	6	160
Number of data point in field data	197	160	167	11	535

The number of investigated records was 197 for fish species inhabiting seawater and mudflats and 160 for species inhabiting only freshwater (Table 1). However, when considering the availability of length data and length-weight coefficients for each species, the number of usable data points was reduced to 114 for seawater and mudflat species and 131 for freshwater species (Table 2 in S1 Appendix). The number of data points for each species within each dataset is provided in the Supporting information.

**Fig 2:**
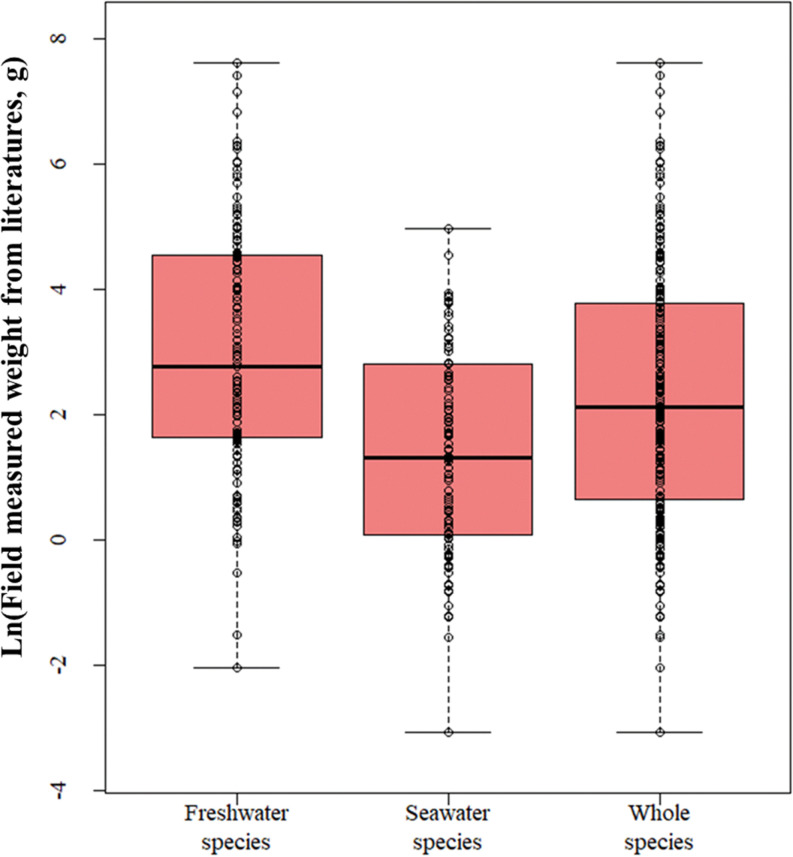
Box plot for Ln- recorded field weight of grouped species.

The distributions of individual biomass for 131 freshwater, 114 seawater, and 245 species from the database are presented as box plots (Fig 2). Since biomass values range widely, from less than 1 g for small species to over 1000 g for larger species such as carp, applying a simple box plot would result in skewness due to scale differences. Therefore, a logarithmic scale was applied to the boxplots (Fig 2). To detect outliers within each dataset, the 1.5 times the interquartile range (IQR) method was used to detect outliers in each dataset. No outliers were found in any of the datasets when this criterion was used (Fig 2). The range of field measured weights from literature for each dataset is 0.1299 g/inch. to 2024.4 g/ind. for freshwater species, and 0.0471 g/ind. to 143.3 g/ind. for seawater species and 0.0471 g/ind. to 2024.4 g/ind. in the combined species groups (Fig 2).

The length-weight coefficients for each species were collected from FishBase [40], with only data from studies conducted in Korea on accurately identified species. The length–weight coefficients collected for each species are presented in tabular form (Table 1 in S1 Appendix).

The General Total Length (GTL) representing each species were recorded based on data from FishBase, online databases, and field guides. For each species, length was recorded as either the average of the minimum and maximum lengths reported from these sources or the explicitly stated average length.

### Data processing and analysis of concordance between estimated and measured field weight from literature

**Fig 3:**
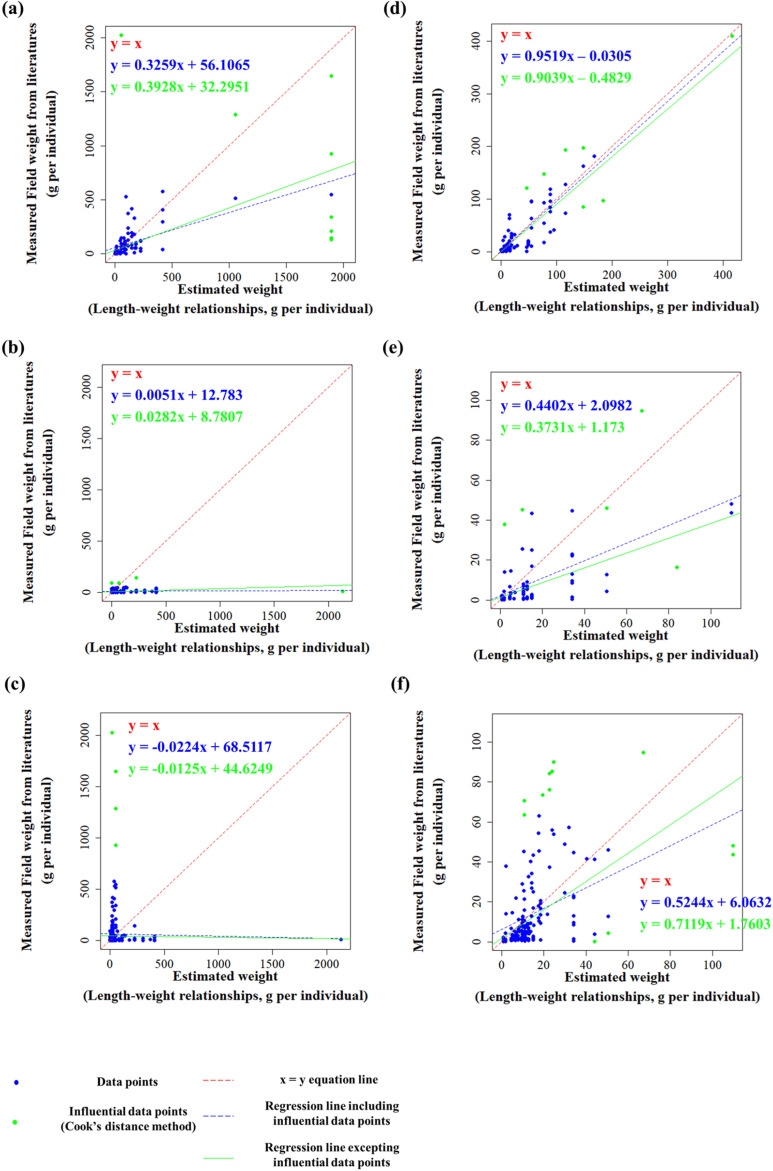
Graph plotting for dataset group (freshwater, seawater, whole species) with outlier processing. (a) Targeted only freshwater species with no exception on outlier. (b) Targeted only seawater species with no exception on outlier. (c) Targeted whole species with no exception on outlier. (d) Targeted only freshwater species without outlier with Q > 6. (e) Targeted only seawater species without outlier with Q > 6. (f). Targeted whole species without outlier with Q > 6.

The results presented in Fig 3 illustrate the comparison between the estimated and measured field biomasses from literatures for freshwater and seawater species with each data point analyzed (Fig 3). The estimated weights, calculated using the GTL and representative constants of LWRs from the literature, are plotted on the x-axis, whereas the field weights reported in the literature are plotted on the y-axis. A Q value was calculated for each data point and data with Q >  6 were removed from the dataset for analysis. Consequently, each group was divided into two datasets: the original dataset and a modified dataset, excluding data points with Q >  6. The analysis was conducted across six datasets categorized by freshwater species, seawater species, and all species combined for the original and Q > 6 eliminated datasets.

In each graph, Cook’s distance was used to identify the influential data points for trend analysis, and separate trend lines were presented for cases in which these data points were included or excluded. The blue line represents the first-order linear regression line calculated using the entire dataset, including influential data points (Fig 3). The green line represents the first-order linear regression line calculated after removing the influential data points identified by the Cook’s distance. In addition, the red dashed line represents the y =  x line, which serves as a reference line where the estimated weight equals the measured field biomasses from literature, indicating an ideal alignment between the two values (Fig 3).

In panel (a), for freshwater species without removing the Q >  6 values, the slope of the trend line before removing the influential points was 0.3259; after removing them, the slope slightly increased to 0.3928 (Fig 3a). In panel (b), for seawater species without removing Q >  6, the slope of the trend line before removing the influential points was 0.0051; after removal, the slope increased slightly to 0.0282 (Fig 3b). In panel (c), for the whole dataset of freshwater and seawater species without removing Q >  6 values, the slope of the trend line was -0.0224 before removing the influential points, and after removal, it slightly increased to -0.0125 (Fig 3c).

In contrast, in Panel (d), for freshwater species with Q >  6 values removed, the slope of the trend line was 0.9519 before removing the influential points and decreased slightly to 0.9039 after removal (Fig 3d). In panel (e), for seawater species with Q >  6 values removed, the slope decreased from 0.4402 to 0.3731 after removing influential points (Fig 3e). In panel (f), for the entire dataset of freshwater and seawater species with Q >  6 values removed, the slope of the trend line increased from 0.5244 to 0.7119 after removing influential points (Fig 3e). Overall, the change in slope after removing the influential points varied depending on the dataset.

### Analysis of the coefficient of determination (R²) for each dataset comparing estimated and measured field weight from literatures

**Fig 4:**
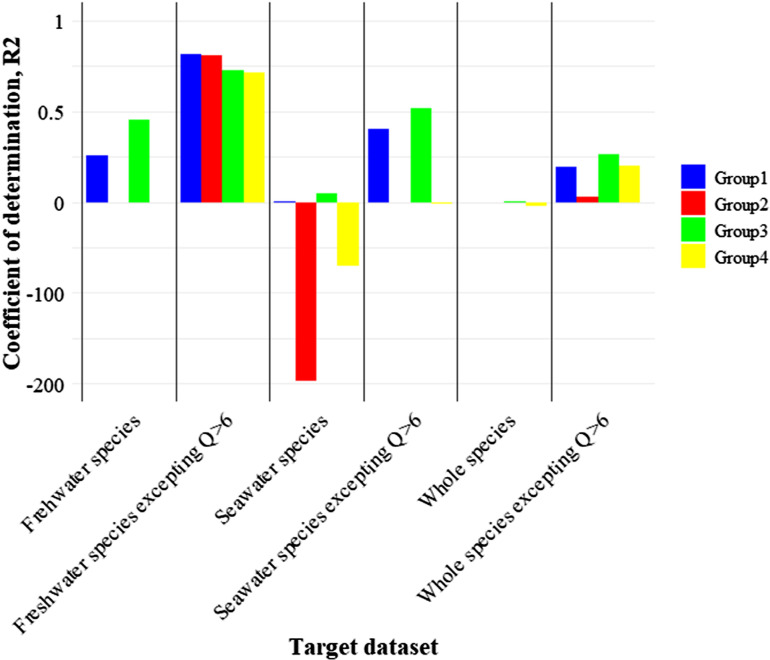
Chart for coefficient of determination (R^2^) of each dataset.

In Fig 4, the correlation between the estimated and measured field biomasses from literatures for the six dataset groups, as shown in the chart, was evaluated by calculating the coefficient of determination (R²) through statistical processing for Group 1, 2, 3, and 4. For Group 1, R² represents the value derived by applying a first-order linear regression to the data points (Fig 4). Group 2 presents the R² values for the data points with respect to the y =  x equation. Group 3 represents the R² values after removing influential points, as identified by Cook’s distance, and applying first-order linear regression to the remaining data points. Finally, Group 4 shows the R² value for the data points with respect to the y =  x equation after removing the influential points based on Cook’s distance (Fig 4).

For freshwater species, R² for the regression of the dataset was 0.2583, and after removing influential points, it increased to 0.4558 (Fig 4). The R² for the y =  x equation was -0.8989, improving to -0.6644 after removing influential points, indicating an increase in R² after the removal of influential points (Fig 4). For the dataset of freshwater species with Q >  6 removed, the regression R² was 0.8140, decreasing to 0.7292 after removing the influential points, whereas the R² for the y =  x equation was 0.8109, decreasing to 0.7133 after removing the influential points (Fig 4).

For seawater species, the regression R² of the dataset was 0.0047, increasing to 0.0474 after removing influential points, while the R² for the y =  x equation was -197.24, improving to -70.682 after removing influential points (Fig 4). For the dataset of seawater species with Q >  6 values removed, the regression R² was 0.4049, increasing to 0.5161 after removing influential points, and the R² for the y =  x equation was -0.4748, decreasing to -1.514 after removing influential points (Fig 4).

For the entire species group, the regression R² of the dataset was 0.00005, increasing to 0.0008 after removing the influential points, whereas the R² for the y =  x equation was -0.98952, further decreasing to -4.959 after removing the influential points (Fig 4). For the entire species dataset with Q >  6 values removed, the regression R² was 0.1914 and increased to 0.2606 after removing the influential points, whereas the R² for the y =  x equation was 0.02896 and increased to 0.1994 after removing the influential points (Fig 4).

In summary, the dataset with the highest regression R² was the group of freshwater species with Q >  6 values removed, and the group with the highest R² for the y =  x equation was the freshwater species group with Q >  6 values removed. In contrast, the seawater species and whole species groups exhibited lower R² and R² values in the y =  x equation. Additionally, the RMSE and MAE values for evaluating the efficiency of the regression model and y =  x model for each dataset group are presented in the Supplementary Materials (Table 1 in S1 Appendix).

## Discussions

The aim of this study was to assess the applicability of biomass estimates for fish species in Korea by utilizing the GTL derived from literature and representative LWR coefficients. This study compared the concordance between estimated and actual measured field biomasses from literature for both freshwater and seawater species.

As shown in Fig 3, the effect of outlier removal varies depending on the dataset for each fish group. In some panels, such as (b), (c), and (d), the slope and intercept of the trend line do not change significantly after removing influential data points, whereas in other panels, such as (a), (e), and (f), the slope and intercept change considerably (Fig 3). This suggests that specific data points have a substantial effect on the overall analysis results for certain species. In datasets where the measured field biomasses from literatures or estimated biomasses values exhibited extreme outliers, the regression slope was heavily influenced, and the removal of influential points proved effective [41,42,50].

Comparing the slope and interceptive values across the panels in Fig 3, Panels (a), (b), and (c), which include Q >  6 data points, show slopes that deviate significantly from the ideal trend line, y =  x (Fig 3a, b, and c). Because Q is calculated based on the absolute value of y - x, datasets containing outliers corresponding to high Q values exhibit large errors when the relationship y =  x [41]. Consequently, as observed in Panels (d), (e), and (f), removing data points with Q >  6 improves the alignment of the regression line with y =  x.

Although panel (e) for seawater species still does not represent an ideal trend line after removing Q >  6 data points, panels (d) and (f) for freshwater and whole species show an improved alignment with the y =  x line (Fig 3e, d, and f). In panel (d), the slope of the regression line is 0.9519, which is close to the y =  x line, with a small intercept (Fig 3d). However, after removing the influential points to improve data reliability, the slope decreased slightly to 0.9039, moving further from the ideal trend (Fig 3d). In panel (f), the slope before removing the influential points is 0.5244, significantly deviating from 1; however, after their removal, the slope improves to 0.7119, moving closer to 1 (Fig 3f). This increase in the slope towards y =  x after removing Q >  6 points indicated that the regression line became more aligned with the ideal trend.

The increase in the slope after removing Q >  6 values suggests that the removed data points likely had much higher estimated values than the measured field biomasses from literature. These removed points were often small individuals, such as juveniles, whose measured field biomasses from literatures were much smaller than the average, leading to their classification as outliers [20,51,52]. Therefore, the presence of juvenile fish appears to be a significant factor contributing to inaccuracies in general biomass estimation.

In Fig 3, the datasets that display the trend line closest to the y =  x line, representing the agreement between the estimated and measured field biomasses from literatures, correspond to the datasets for freshwater species and whole species with Q >  6 values removed, as shown in panels (d) and (f) (Fig 3d and f). In contrast, the regression graph for seawater species followed a trend that was difficult to explain using the y =  x line, indicating that the estimated biomasses derived from the literature-based GTL and representative LWR coefficients did not match the actual measured field biomasses from literatures for coastal fish species in Korea (Fig 3b and e). Therefore, the biomass estimation method based on the literature-derived GTL and representative LWR coefficients demonstrates a more suitable correlation for freshwater species than for seawater species in Korea [20,34].

In Fig 4, the relationship between the predicted and measured field biomasses from literatures in this study was evaluated using the R² value with respect to the y =  x line, as this represents the true correspondence between the two values, and indicates the validity of the biomass estimation method. Thus, the R² values assessed in Groups 2 and 4 reflect the degree of agreement between the estimated and measured field biomasses from literatures in each dataset, whereas the R² values in Groups 1 and 3 represent the consistency of trends within the dataset itself. Among the six datasets analyzed, the dataset for freshwater species with Q >  6 values removed was the only one in which the biomass estimation methodology was appropriately applied (Fig 4). In this dataset, the R² value for the y =  x equation was 0.8109, and after removing the influential points, it decreased slightly to 0.7133; however, the estimates generated by the length-weight relationship still showed a meaningful fit with the biomass recorded in the literature. In comparison, the R² value for the y =  x equation in the dataset for freshwater species without Q removal was -0.899, and after removing influential points, it improved to -0.664, showing a significant correlation with the y =  x line after removing Q outliers (Fig 4).

The reason for this improvement is that, as previously mentioned, the measured field biomasses from literatures for freshwater species in this study included a significant number of juveniles [20]. By removing the outliers corresponding to Q values greater than six, the alignment between the predicted biomass calculated from the general body length and the biomass recorded in the literature improved [41]. Notably, 32 data points (approximately 25% of the total) were excluded because Q >  6 (Fig 4). Furthermore, the R² values for the datasets with simple linear regression were generally higher than those for the y =  x equation across all the datasets, indicating a general correlation between the measured field biomasses from literatures and estimated biomasses. However, the R² values for the y =  x equation, which represent perfect agreement between the estimated and measured field biomasses from literatures, were very low for all datasets except for that for freshwater species with Q >  6 removed (Fig 4). The seawater species dataset produced negative R² values, indicating a lower level of agreement than expected from the simple average biomass estimates. The applicability of length-weight coefficients appears to vary across different environments for fish, as demonstrated in several studies [20,34,53].

The study about length-weight relationship of *Mystus tengara* in pond and river habitat in India showed r^2^ of length-weight relationship for *Mystus tengara* living in river was higher than *Mystus tengara* living in pond. However, Pearson’s correlation between habitat and condition factor shows no significant correlation in same study [54]. Similarly, a study on the length-weight relationship of *Tenualosa ilisha* in Bangladesh rivers (freshwater) and the Bay of Bengal (seawater) revealed no significant differences in b values between samples obtained from riverine and seawater habitats [55]. However, the size range and weight range of the target species were found to be higher in the Bay of Bengal (seawater) compared to the Bangladesh rivers (freshwater) [55]. However, this is a study applied to sampling results of a single species. What can be inferred from this is that the length-weight constants a and b for a single species do not vary significantly according to habitat, but individuals inhabiting seawater may generally exhibit larger sizes [55].

This can explain why the applicability of the standard biomass estimation methodology for freshwater species in this study appears to be higher than that for seawater species. Although the applicability of length-weight constants may not differ significantly between habitats, seawater samples are generally likely to be larger and have a wider size distribution compared to river samples [54,55]. As a result, the General Total Length (GTL) used in this study may be inadequate in representing the wide range of sizes for each species in seawater, even if it was recorded based on rivers or seawater standards for the same species. Therefore, while the applicability of length-weight constants may not differ greatly between rivers and seawater, it appears that the applicability of representative lengths (GTL) for each species differs between seawater and freshwater environments

Therefore, the biomass estimates derived from the GTL-based methodology in this study, which used coefficients for LWRs obtained from the literature on fish species in Korea, were applicable only to freshwater species.

Based on the results of this study, when considering the relationship and coefficient of determination between the estimated and measured field biomasses from literature, it is evident that applying literature-based biomass estimation for fish species in Korea is appropriate only when the species has been confirmed to inhabit freshwater environments.

## Conclusion

The purpose and originality of this study was to estimate the biomass of fish species inhabiting freshwater and seawater in Korea using literature-based General Total Length (GTL) and Length-Weight Relationships (LWRs), and to evaluate the accuracy of the estimates by comparing them with measured field biomasses from literature. The applicability of the general biomass estimates calculated using LWRs and GTL from the literature was compared across different fish groups. When comparing freshwater species, seawater species, and a combined group of both, only the freshwater species group showed a close correlation between the estimated and measured field biomasses from literatures, with R² values of 0.8109 before removing influential points and 0.7133 after their removal, demonstrating significant explanatory power. In contrast, the applicability of the seawater species group was minimal with negative R² values. Considering the coefficient of determination, the biomass estimates derived from the GTL and LWRs were found to be appropriate only for species that inhabit freshwater in Korea. Based on these results, when performing biomass estimates using data from the literature instead of measured field biomasses from literatures, it is essential to confirm the habitat of the species and limit the estimates to freshwater species to obtain accurate results. This study is expected to contribute to the generation of fundamental data on biomass that can be used in future studies on ecosystem indicators and ecological modeling. Furthermore, this study highlights the need for future research to expand beyond the existing species data and explore biomass estimation through extrapolation among species.

## Supporting information

S1 Appendix Table dataset for supporting information. (DOCX)

S2 Appendix Dataset for measured and estimated dry weight. (XLSX)
