# Supplementary material for: Comparing efficiency of literature-based estimation of general biomass using Length-Weight Relationships (LWRs) between freshwater and seawater fishes
Source: PLoS One. 2025 Jun 3;20(6):e0321571. doi: 10.1371/journal.pone.0321571 (PMC12132944; doi:10.1371/journal.pone.0321571)
Supplement: S1 Appendix — (DOCX) [file pone.0321571.s001.docx]

# Supplementary materials

Table 1 Statistical analysis for each group of dataset

| **Group name** | **Freshwater** | **Freshwater Q exception** | **Seawater** | **Seawater Q exception** | **Whole** | **Whole Q exception** |
| --- | --- | --- | --- | --- | --- | --- |
| Number of data points before excepting influential point | 131 | 99 | 114 | 86 | 245 | 181 |
| Number of Cook's Influential point | 8 | 7 | 5 | 6 | 4 | 15 |
| Number of data points after excepting influential point | 123 | 92 | 109 | 80 | 241 | 166 |
|  | **Coefficient for the regression equation for data points before excepting influential point** | | | | | |
| **Group name** | **Freshwater** | **Freshwater Q exception** | **Seawater** | **Seawater Q exception** | **Whole** | **Whole Q exception** |
| R2 | 0.25830 | 0.81400 | 0.00470 | 0.40490 | 0.00005 | 0.19140 |
| p-value | <0.001 | <0.001 | NaN | <0.001 | 0.7340 | <0.001 |
| RMSE | 239.55270 | 25.77750 | 21.64870 | 12.57930 | 209.98270 | 18.26710 |
| MAE | 106.46670 | 16.37010 | 14.51060 | 7.57230 | 89.92010 | 12.45720 |
|  | **Coefficient for the y = x equation for data points before excepting influential point** | | | | | |
| **Group name** | **Freshwater** | **Freshwater Q exception** | **Seawater** | **Seawater Q exception** | **Whole** | **Whole Q exception** |
| R2 | -0.89893 | 0.81090 | -197.23795 | -0.47489 | -0.98952 | 0.02896 |
| p-value | <0.001 | <0.001 | NaN | <0.001 | <0.001 | 0.6988 |
| RMSE | 383.29099 | 25.98942 | 305.52635 | 19.80412 | 296.25161 | 20.01779 |
| MAE | 131.35213 | 16.40165 | 94.94726 | 12.26295 | 98.62364 | 12.45973 |
|  | **Coefficient for the regression equation for data points after excepting influential point** | | | | | |
| **Group name** | **Freshwater** | **Freshwater Q exception** | **Seawater** | **Seawater Q exception** | **Whole** | **Whole Q exception** |
| R2 | 0.45580 | 0.72920 | 0.04740 | 0.51610 | 0.00080 | 0.26060 |
| p-value | <0.001 | <0.001 | 0.02299 | <0.001 | 0.6720 | <0.001 |
| RMSE | 88.28520 | 19.40470 | 19.96730 | 7.89120 | 93.79450 | 12.32990 |
| MAE | 52.91020 | 12.79090 | 10.76920 | 4.67990 | 53.61160 | 8.22910 |
|  | **Coefficient for the y = x equation for data points after excepting influential point** | | | | | |
| **Group name** | **Freshwater** | **Freshwater Q exception** | **Seawater** | **Seawater Q exception** | **Whole** | **Whole Q exception** |
| R2 | -0.66439 | 0.71327 | -70.68178 | -1.51413 | -4.95944 | 0.19938 |
| p-value | <0.001 | <0.001 | NaN | NaN | NaN | 0.01002 |
| RMSE | 154.40037 | 19.96727 | 122.70394 | 17.98606 | 229.05668 | 12.83007 |
| MAE | 57.36015 | 13.01847 | 58.56122 | 10.99669 | 76.56594 | 8.71931 |

Table 2 The number of data point in dataset for each species

| **Freshwater** | | **Seawater** | |
| --- | --- | --- | --- |
| Name (Species) | Number of data point | Name (Species) | Number of data point |
| *Acheilognathus lanceolatus* | 3 | *Argyrosomus argentatus* | 4 |
| *Acheilognathus rhombeus* | 1 | *Chaeturichthys hexanema* | 1 |
| *Acheilognathus yamatsutae* | 3 | *Cryptocentrus filifer* | 4 |
| *Anguilla japonica* | 6 | *Cynoglossus abbreviatus* | 1 |
| *Carassius auratus* | 5 | *Cynoglossus joyneri* | 9 |
| *Carassius cuvieri* | 3 | *Engraulis japonicus* | 7 |
| *Channa argus* | 2 | *Gymnogobius heptacanthus* | 11 |
| *Coreoperca herzi* | 4 | *Hexagrammos otakii* | 6 |
| *Cyprinus carpio* | 7 | *Inimicus japonicus* | 1 |
| *Erythroculter erythropterus* | 1 | *Limanda yokohamae* | 3 |
| *Hemibarbus labeo* | 6 | *Liparis tanakai* | 1 |
| *Hemibarbus longirostris* | 4 | *Muraenesox cinereus* | 1 |
| *Hemiculter eigenmanni* | 1 | *Pampus argenteus* | 2 |
| *Leiognathus nuchalis* | 4 | *Paralichthys olivaceus* | 4 |
| *Microphysogobio yaluensis* | 4 | *Pholis nebulosa* | 13 |
| *Micropterus salmoides* | 3 | *Pterogobius elapoides* | 6 |
| *Misgurnus anguillicaudatus* | 3 | *Repomucenus lunatus* | 6 |
| *Misgurnus mizolepis* | 1 | *Repomucenus valenciennei* | 1 |
| *Odontobutis interrupta* | 3 | *Rudarius ercodes* | 4 |
| *Odontobutis platycephala* | 2 | *Scomberomorus niphonius* | 2 |
| *Opsariichthys uncirostris* | 4 | *Sebastes inermis* | 1 |
| *Plecoglossus altivelis* | 3 | *Sphyraena pinguis* | 2 |
| *Pseudobagrus fulvidraco* | 7 | *Taenioides rubicundus* | 9 |
| *Pseudobagrus koreanus* | 2 | *Thryssa adelae* | 3 |
| *Pseudogobio esocinus* | 4 | *Thryssa kammalensis* | 8 |
| *Pseudorasbora parva* | 4 | *Trachurus japonicus* | 1 |
| *Pungtungia herzi* | 4 | *Trichiurus lepturus* | 3 |
| *Rhinogobius brunneus* | 4 |  |  |
| *Rhynchocypris oxycephalus* | 1 |  |  |
| *Sarcocheilichthys nigripinnis* | 3 |  |  |
| *Sarcocheilichthys variegatus* | 3 |  |  |
| *Silurus asotus* | 4 |  |  |
| *Siniperca scherzeri* | 5 |  |  |
| *Squalidus gracilis* | 4 |  |  |
| *Squalidus multimaculatus* | 1 |  |  |
| *Tridentiger brevispinis* | 1 |  |  |
| *Tridentiger obscurus* | 2 |  |  |
| *Zacco koreanus* | 5 |  |  |
| *Zacco platypus* | 4 |  |  |
| **Total** | 131 | Total | 114 |
